# Robust CD8^+^ T cell responses induced by an mRNA-LNP vaccine encoding rat HER2 extracellular domain confer prophylactic tumor protection

**DOI:** 10.3389/fimmu.2026.1737558

**Published:** 2026-04-01

**Authors:** Kexin Li, Xiaoya Li, Cong Zhang, Suli Dai, Lei Li, Jun Wang, Hongtao Zhang, Lianmei Zhao

**Affiliations:** 1 Research Center, The Key Laboratory of Tumor Prevention and Precise Diagnosis & Treatment in Hebei Province, the Fourth Hospital of Hebei Medical University, Shijiazhuang, Hebei, China; 2 Department of Clinical Laboratory, Shandong Cancer Hospital and Institute, Shandong First Medical University and Shandong Academy of Medical Sciences, Jinan, Shandong, China; 3 Department of Radiation Oncology, Hebei Clinical Research Center for Radiation Oncology, The Fourth Hospital of Hebei Medical University, Shijiazhuang, Hebei, China; 4 Hangzhou CytoCan Biotech, Hangzhou, Zhejiang, China

**Keywords:** breast cancer, HER2, immunotherapy, lipid nanoparticle (LNP), mRNA vaccine

## Abstract

**Background:**

HER2-positive cancers present challenges of drug resistance, toxicity, and immune tolerance. HER2-targeted peptide vaccines have failed clinically, while nucleoside-modified mRNA-lipid nanoparticle (mRNA-LNP) vaccines show promise for robust antitumor immunity. This study utilized heterologous immunity (rat HER2 extracellular domain, rHER2 ECD) to circumvent HER2 tolerance, developing a safe mRNA-LNP vaccine and exploring its synergy with anti-PD-1 therapy.

**Methods:**

We synthesized N1-methyl-pseudouridine (m1ψ)-modified mRNAs, encoding either rHER2 ECD alone (rHER2 ECD mRNA-LNP) or rHER2 ECD fused to murine IFNγ (rHER2 ECD-IFNγ mRNA-LNP), and then encapsulated them in LNPs via microfluidic mixing. BALB/c mice were immunized on days 1, 15, 29, then challenged with HER2-overexpressing 4T1 (4T1-HER2) cells 2 weeks post-final immunization. Efficacy was assessed by tumor growth, immune responses via flow cytometry, ELISA, ELISpot and cytotoxicity assays, safety via body/organ weight and serum markers.

**Results:**

HER2 ECD mRNA-LNPs exhibited >80% encapsulation efficiency and narrow particle size distribution. As monotherapy, rHER2 ECD mRNA-LNPs induced high anti-HER2 antibody titers, polyfunctional CD8^+^ T cells (IFNγ^+^/TNFα^+^), and durable memory T cells, achieving 87.0% tumor inhibition. This inhibition rate was significantly higher than that of the rHER2 ECD-IFNγ mRNA-LNP variant, which achieved 21.9% tumor inhibition and induced anti-IFNγ neutralizing antibodies. Combination with anti-PD-1 monoclonal antibody (mAb) further enhanced tumor inhibition, with 2/6 tumors failing to establish. This synergy was driven by tissue-resident memory T (T_RM_) cell enrichment in the tumor microenvironment (TME), which reached 15.2% in CD4^+^ T cells and 12.9% in CD8^+^ T cells. No systemic toxicity was observed.

**Conclusion:**

The rHER2 ECD mRNA-LNP effectively circumvents HER2-specific immune tolerance. As a potent and safe platform, it induces robust coordinated humoral and cellular immunity and significantly enhances checkpoint blockade efficacy via the enrichment of T_RM_ cells in the TME, which underscores its promising potential for the treatment of HER2-positive cancers.

## 1 Introduction

HER2, a critical member of the human epidermal growth factor receptor family, is overexpressed in various solid tumors, including breast cancer (1–3), gastric cancer (4, 5), esophageal cancer (6), cholangiocarcinoma (7), and lung cancer (8). The receptor consists of an extracellular domain (ECD), a transmembrane domain (TMD), and an intracellular tyrosine kinase domain (TKD), with oncogenic mutations primarily occurring in the kinase or extracellular domains (9). Although targeted therapies such as monoclonal antibodies (mAbs) and antibody-drug conjugates (ADCs) have improved clinical outcomes, their effectiveness is often compromised by primary and acquired resistance (10, 11). Moreover, these therapies and conventional chemoradiotherapy are associated with significant toxicities and fail to achieve durable remissions in most patients (12). Critically, in early-stage HER2-positive breast cancer, conventional modalities often fail to eliminate minimal residual disease (MRD) after curative resection, leaving it a key driver of recurrence (13). This enduring clinical challenge highlights the pressing necessity for innovative strategies that can elicit robust and durable antitumor immunity with enhanced safety profiles.

HER2 remains a well-validated target due to its tumor-specific overexpression and validated role in oncogenesis (14). Despite decades of research into HER2-targeted peptide vaccines, including E75 (15), GP2 (16), AE37 (17), NeuVax (18), Adagloxad Simolenin (19) and HER-Vaxx (IMU-131) (20), these efforts have consistently resulted in measurable immune responses but have failed to achieve clinical approval. This shortfall is primarily attributed to challenges such as low antigen delivery efficiency, transient immune responses, and Major Histocompatibility Complex (MHC) restriction. The groundbreaking success of nucleoside-modified messenger RNA (mRNA) vaccines encapsulated in lipid nanoparticles (LNPs) against SARS-CoV-2 has reinvigorated the field of cancer immunotherapy (21, 22). mRNA-LNP vaccines present distinct technical advantages, such as efficient cellular delivery for endogenous protein production (23), the induction of coordinated humoral and cellular immune responses (24), avoidance of genomic integration (25), and enhanced stability through modifications like N1-methyl-pseudouridine (m1ψ), which reduce innate immunogenicity while enhancing translational efficiency (26, 27). These attributes support mRNA-LNP as a promising strategy for the treatment of advanced solid tumors, as corroborated by accumulating preclinical and clinical evidence (28–30). However, both peptide and mRNA-LNP vaccines encounter similar clinical challenges, with the immunosuppressive tumor microenvironment (TME), which is related to the upregulation of PD-L1 in response to vaccine-induced interferon gamma (IFNγ) signaling, representing a significant obstacle that hinders vaccine-induced antitumor immunity (31). Other factors including tumor heterogeneity and antigen escape also contribute to the lack of robust objective clinical responses observed in the majority of patients treated with either type of vaccine (32, 33). To address these unmet challenges, the combination of mRNA vaccines with PD-1/PD-L1 inhibitors has emerged as a promising strategy (28). This approach aims to leverage checkpoint blockade to counteract vaccine-induced immune suppression in TME, while harnessing the potent immunostimulatory properties of mRNA-LNPs.

To mitigate immune tolerance and enhance therapeutic outcomes, we developed an mRNA-LNP vaccine encoding the ECD of rat HER2. This design employs the principle of heterologous immunity, wherein antigens derived from a different species (rat) disrupt immune tolerance and enhance immune responses against self-proteins such as HER2 (34, 35). Notably, the rat HER2 sequence elicits a more robust Type 1 helper T lymphocyte (Th1) response compared to its human equivalent in preclinical models (36). The high degree of rat-human homology ensures translational relevance, while the use of rat antigens in mouse models provides a controlled syngeneic system for investigating adaptive immunity, including HER2-specific T-cell receptor repertoires.

In this study, we innovatively incorporated nucleoside modification and an optimized LNP formulation to enhance the translation efficiency and *in vivo* delivery of mRNA. To assess preventive efficacy against HER2-positive tumors, we evaluated several strategies: rHER2 ECD mRNA-LNP alone, a fusion construct co-expressing rat HER2 ECD and mouse IFNγ to enhance antigen presentation and T cell activation, and a combination of rat HER2 ECD mRNA-LNP with PD-1 mAb to inhibit immune suppression. Vaccination, particularly with combined strategies, induced potent antibody production, robust CD8^+^ T cell responses, and memory T cell formation, with only mild adverse effects observed. This work established the rHER2 ECD mRNA-LNP platform as a promising preventive strategy against HER2-positive cancers, offering essential immunological insights and a robust foundation for clinical translation.

## 2 Methods

### 2.1 mRNA production

DNA sequences encoding the ECD of rat HER2, as well as the rat HER2 ECD fused in-frame with murine IFNγ via a polyglycine (Poly-G) linker, were synthesized. Both constructs included an N-terminal signal peptide to direct protein secretion. These sequences, flanked by optimized 5’ and 3’ untranslated regions (UTRs) (General Bio, Chuzhou, China), were cloned into the pcDNA 3.1(+) plasmid. Primer synthesis and sequence verification were conducted by Sangon Biotech (Shanghai, China). The plasmids were linearized using the EcoRI restriction enzyme (#FD0274, Thermo Scientific, USA) and served as templates for *in vitro* transcription employing T7 RNA polymerase (#E2040S, New England Biolabs, USA). During transcription, m1ψ-5’-triphosphate (#N-1081-10, TriLink, USA) was incorporated in lieu of uridine-5’-triphosphate to produce nucleoside-modified mRNA. The resulting mRNAs were capped using the m7G capping kit (#M2080S, New England Biolabs, USA) in conjunction with 2’-O-methyltransferase (#M0366S, New England Biolabs, USA) to form Cap1 structures. Polyadenylation was performed using E. coli Poly(A) Polymerase (#M0276L, New England Biolabs, USA). The synthesized mRNAs underwent purification with the Monarch RNA Cleanup Kit (#T2050L, New England Biolabs, USA) to eliminate double-stranded RNA contaminants. The integrity of the mRNAs was evaluated through agarose gel electrophoresis. All mRNA samples were aliquoted and stored at -80 °C. Nucleotide sequences are summarized in **Supplementary Table 1**.

### 2.2 Generation and characterization of mRNA-LNPs

(4 - (N,N - dimethylamino) butyl) methyl - (cis - 9 - octadecenyl) - (9Z,12Z) - octadecadien - 1 - amine (Dlin-MC3-DMA; AVT, China), 1,2 - Distearoyl - sn - glycero - 3 - phosphocholine (DSPC; AVT, China), cholesterol (CHOL; #C8667-1G, Sigma, Germany) and 1,2-Dimyristoyl-sn-glycerol-methoxy-polyethylene glycol 2000 (DMG-PEG2000; AVT, China) were dissolved in ethanol at a molar ratio of 50:10:38.5:1.5 (**Table 1**). The ethanol phase and aqueous phase were merged at a 3:1 volume ratio using a Nano S microfluidic (Unigen Inc., Suzhou, China). This mixture was then added to 50 mM citrate buffer (pH = 4), where mRNA encapsulation occurred via a self-assembly process at a mRNA-to-LNP mass ratio of 0.06 (37). The resultant mRNA-LNP complexes underwent dialysis against PBS (pH 7.4) using a D-Tube™ Dialyzer Maxi (#71509-3, Millipore, USA) to eliminate residual ethanol and facilitate buffer exchange. The final mRNA-LNP formulations were either utilized immediately or stored at 4 °C. Encapsulation efficiency was assessed via the RiboGreen assay (#R11491, Invitrogen, USA). For this purpose, mRNA-LNPs were diluted in TE buffer to measure free mRNA or in TE buffer containing 2% Triton X-100. Fluorescence intensity was measured using a multimode microplate reader following incubation with the RiboGreen reagent. Encapsulation efficiency was calculated using the formula:Encapsulation efficiency (%) = total mRNA (mRNA_total_) - dissociated mRNA (mRNA_free_)/mRNA_total_ × 100%. The average diameter and span were assessed utilizing the NanoCoulter instrument (RESUNTECH, Shenzhen, China).

**Table 1:** Formulation of LNP.

Component	Ratios	C (mM)
Dlin-MC3-DMA	50	12.5
DSPC	10	
CHOL	38.5	
DMG-PEG2000	1.5	

### 2.3 Cell lines and cell culture

The 4T1-HER2 cell line, characterized by high HER2 expression and provided by HankeMab (Suzhou, China), was developed through HER2 plasmid transfection. The cells were cultured in RPMI 1640 medium, supplemented with 10% fetal bovine serum (FBS), penicillin-streptomycin (50 U/mL), and G418 (0.5 mg/mL; #G8161, Solarbio, China), maintained at 37 °C in a 5% CO_2_ atmosphere. Routine monitoring for mycoplasma contamination was performed using PlasmoTest (InvivoGen). Authentication of the cell line involved verification of the original source, expansion under quarantine conditions to ensure purity before establishing progenitor stocks, and pre-experimental screening to confirm mycoplasma negativity (PlasmoTest), expected morphology, and standard growth kinetics. Cells were utilized only after meeting all quality control criteria.

### 2.4 Animals

All animal procedures were conducted in compliance with the guidelines of the Chinese Association for Laboratory Animal Sciences (CALAS) and were approved by the Institutional Animal Care and Use Committee (IACUC) of the Fourth Hospital of Hebei Medical University under protocol number #IACUC-4th Hos Hebmu-2022062. Female BALB/c mice (4–6 weeks old, weighing 16–18 g) were sourced from Beijing HFK Bio-Technology Co., Ltd. (Beijing, China) and maintained under specific pathogen-free (SPF) conditions at the Experimental Animal Center of the Fourth Hospital of Hebei Medical University, with unrestricted access to food and water. Mice were randomly allocated to various experimental groups to avoid littermate bias.

### 2.5 Tumor implantation and immunization regimens

#### 2.5.1 Immunization

BALB/c mice received intramuscular injections of 10 μg per dose of either rHER2 ECD mRNA-LNP or rHER2 ECD-IFNγ mRNA-LNP into the tibialis anterior muscle on days 1, 15, and 29. Control groups were administered equivalent doses of empty LNP. Blood samples were collected from the orbital venous plexus 14 days following each immunization. For combination therapy, mice were intraperitoneally injected with 100 μg of anti-PD-1 mAb (Clone: RMP1-14; human IgG1 Fc region with L234A/L235A (LALA) mutations; HankeMab, Suzhou, China) or a PBS vehicle every 7 days, for a total of three doses.

#### 2.5.2 Tumor challenge

Two weeks subsequent to the final immunization, the mice were subcutaneously implanted with 2 × 10^5^ 4T1-HER2 cells in 100 μL of sterile PBS into the right flank. Tumor volumes were assessed every 2 days using the formula: Volume = (Length × Width × Height) × 0.5236. Endpoints were established according to CALAS standards, defined as a tumor volume exceeding 1500 mm³ or the presence of ulcerative lesions in progressive tumors. The efficacy of tumor inhibition was assessed at the conclusion of the study. At the treatment endpoint, the tumor inhibition rate (TIR) was calculated as follows: TIR (%)=[1−Mean tumor volume of control group (Vc)/Mean tumor volume of treatment group (Vt)]×100%.

### 2.6 Tissue processing for analysis

Spleens were mechanically homogenized in complete RPMI 1640 medium and filtered through 40 μm cell strainers. Tumor specimens were minced and subjected to enzymatic digestion using the Tumor Dissociation Kit (#130-096-730, Miltenyi Biotec, Germany) in a collagenase solution at 37 °C for 40 minutes, followed by filtration through 70 μm cell strainers (#130-110-916, Miltenyi Biotec, Germany) and washing with complete RPMI 1640 medium. Subsequently, all samples underwent erythrocyte lysis using Erythrocyte Lysate (#BL503A, Biosharp, China) for 5 minutes at 4 °C. The lysis process was halted with the addition of 10 volumes of PBS, followed by centrifugation (spleen: 300 × g; tumor: 500 × g) for 10 minutes. The resulting cell suspensions from both tissues were resuspended in PBS for subsequent analyses.

### 2.7 Intracellular cytokine staining

Single-cell suspensions derived from tumors or spleens (2 × 10^6^ cells/well) were subjected to a 4-hour stimulation using a Leukocyte Activation Cocktail (#550583, BD Biosciences) containing brefeldin A (#555029, BD Biosciences) or were maintained as unstimulated controls (without the activation cocktail). Both experimental conditions were cultured under identical parameters, with data from the unstimulated splenocytes provided in **Supplementary Figure 1**. The cells were incubated with a purified mouse BD Fc Block™ reagent (1 μg/100 μL; #553142, BD Biosciences) for 5 minutes at 4 °C. Subsequently, fixation and permeabilization were conducted using the Transcription Factor Buffer (#562574, BD Biosciences) for 40 minutes. The cells were then stained with optimal concentrations of fluorophore-conjugated antibodies for 40 minutes at 4 °C in the dark. Flow cytometric analysis was carried out using a BD Biosciences instrument, and positive cell populations were quantified utilizing FlowJo v10 software.

### 2.8 Toxicological experiments

Serum samples collected at the study endpoint (day 68) were analyzed for toxicological parameters, including hepatic function markers [aspartate aminotransferase (AST), alanine aminotransferase (ALT), alkaline phosphatase (ALP)], renal function parameters [blood urea nitrogen (BUN), creatinine (CREA), uric acid (UA)], and cardiac injury indicators [lactate dehydrogenase (LDH), creatine kinase (CK), and creatine kinase-MB isoenzyme (CKMB)].

### 2.9 Enzyme-linked immunospot (ELISpot) assay

Splenocytes (2 × 10^5^ cells/well) were stimulated in complete RPMI 1640 medium with or without 2 μg/mL rat HER2 protein (#80079-R08H, Sino Biological) for 12 hours at 37 °C. The production of IFN-γ was quantified using the Mouse Interferon-gamma ELISPOT Kit (#ab64029, Abcam) following the manufacturer’s protocol. After incubation, the plates were washed and sequentially incubated with a biotinylated anti-IFN-γ antibody and a streptavidin-alkaline phosphatase conjugate. Spot-forming units (SFUs) were quantified through automated image analysis.

### 2.10 ELISA

#### 2.10.1 Antibody titer

High-binding enzyme-linked immunosorbent assay (ELISA) plates (#31111B, LABSELECT, China) were coated with 50 μL of either rat HER2 protein or mouse IFNγ protein, each at a concentration of 400 ng/mL in ultrapure water, and incubated overnight at 4 °C. Following incubation, the plates were washed with phosphate-buffered saline containing 0.1% Tween-20 (PBST) and subsequently blocked with a 5% bovine serum albumin (BSA) solution (#A8010, Solarbio, China). Mouse serum samples were then added and incubated at 37 °C for 1 hour. After additional washing steps, the plates were incubated with horseradish peroxidase (HRP)-conjugated Affinipure Goat Anti-Rabbit/Mouse IgG antibodies (#SA00001-1/SA00001-2, Proteintech, China) for 1 hour. Following a final wash, 100 μL of 3,3’,5,5’-tetramethylbenzidine (TMB) substrate (#PR1200, Solarbio, China) was added, and the enzymatic reactions were terminated using an ELISA Stopping Solution (#C1058, Solarbio, China). The optical density (OD) at 450 nm was measured using a FlexA-200 microplate reader.

#### 2.10.2 Cytokine detection

The quantification of HER2 antigen-driven cytokines in the splenocyte supernatants was conducted using Mouse IFN-γ (#EK280), TNF-α (#EK282), IL-2 (#EK202), and IL-4 (#EK204) ELISA Kits (MultiSciences, China), adhering to the manufacturer’s protocols. Splenocytes were stimulated under conditions consistent with the ELISpot assay, specifically at a density of 2 × 10^5^ cells per well in complete RPMI 1640 medium supplemented with 2 μg/mL recombinant rat HER2 protein at 37 °C for 12 hours. Additional experiments involving unstimulated splenocytes and those stimulated for 6 hours were also conducted, with the results detailed in **Supplementary Figure 2**.

### 2.11 Lymphocyte killing assay

1 × 10³ 4T1-HER2 cells were seeded into 96-well plates. Following cell adhesion, 1 × 10^5^ splenic lymphocytes were introduced, and the cultures were incubated at 37 °C. Optical density (OD) values were recorded at 24, 48, and 72 hours using a microplate reader after the addition of 20 μL of CCK-8 reagent to each 200 μL of medium. The cell inhibition rates were calculated using the formula: Inhibition rate (%) = [(OD_s_ - OD_e_)/(OD_s_ - OD_b_)] × 100, where OD_e_ represents the absorbance of the experimental group, OD_s_ denotes the absorbance of the control group, and OD_b_ is the absorbance of the blank wells.

### 2.12 Statistical analysis

Graphical analyses were conducted using GraphPad Prism version 10.3.1. Data are expressed as mean ± SEM from a minimum of three independent experiments. Statistical significance was assessed using two-tailed Student’s t-tests, with one-tailed tests used for select CD4^+^ T cell assays (detailed in figure legends). A p value of less than 0.05 was considered statistically significant. All significant values shown in various figures are indicated as follows: *P < 0.05, **P < 0.01 and ***P < 0.001.

## 3 Results

### 3.1 Robust generation and comprehensive characterization of HER2 ECD mRNA-LNP vaccine formulations

**Figure 1:**
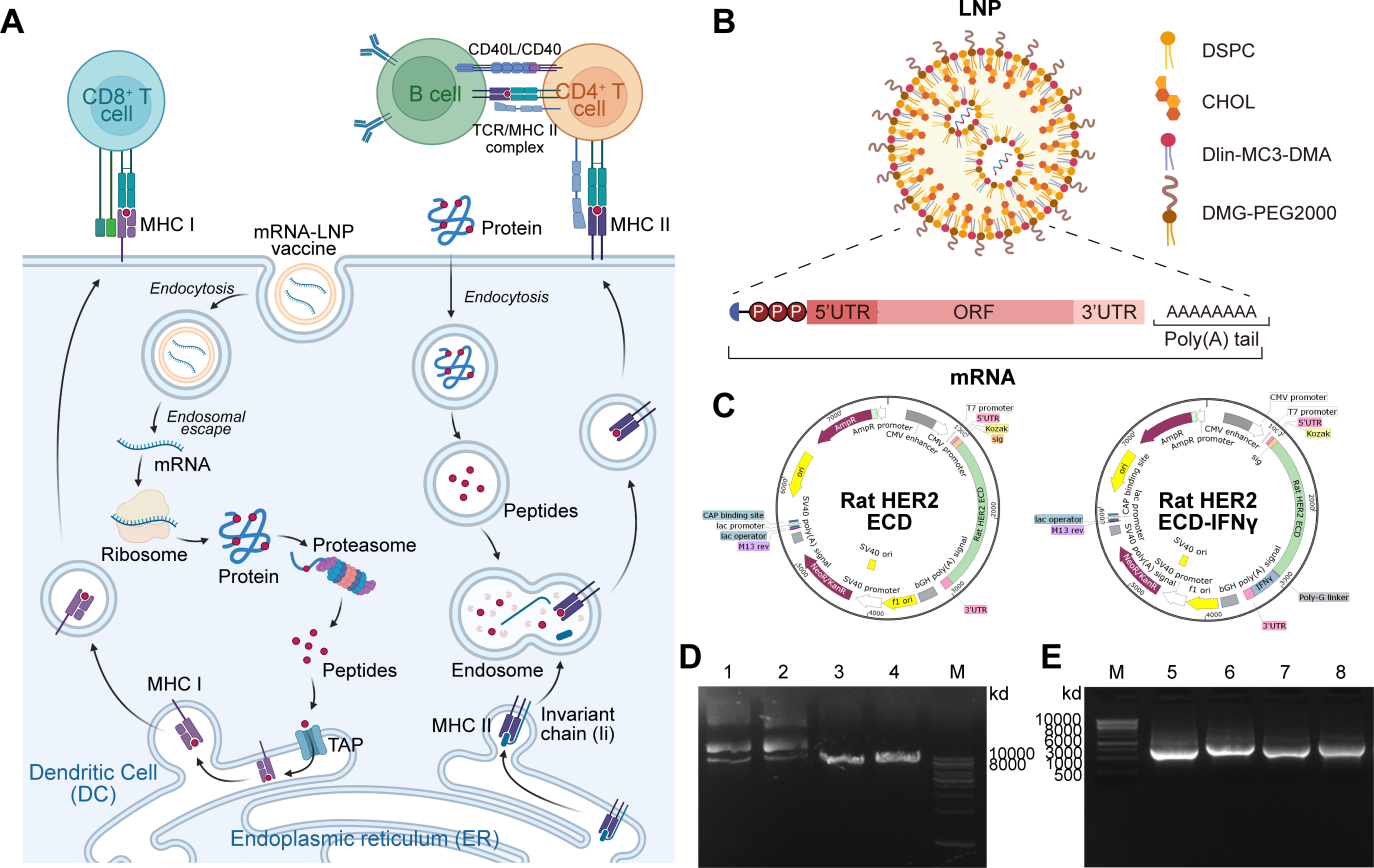
mRNA-LNP vaccine development via integrated mechanism, preparation, and characterization. **(A)** Schematic of the mRNA-LNP vaccine mechanism. Intramuscularly injected mRNA-LNPs are delivered to DCs. The encoded antigen is expressed and processed through two major pathways: the immunoproteasome/MHC class I pathway activates CD8^+^ T cells, while the endosomal/MHC class II pathway activates CD4^+^ T cells. Activated CD4^+^ T cells provide help to B cells for antibody production, orchestrating a coordinated adaptive immune response. **(B)** Schematic microfluidic process of mRNA-LNPs preparation. **(C)** Plasmid map of rat HER2 ECD and rat HER2 ECD-IFNγ. **(D)** Agarose gel electrophoresis of plasmid and linearized plasmid DNA. **(E)** Agarose gel electrophoresis of transcribed mRNA and total mRNA for rat HER2 ECD and rat HER2 ECD-IFNγ. M, 1kb ladder; 1, Plasmids with rat HER2 ECD sequence and 2, rat HER2 ECD-IFNγ sequence; 3, Linearized plasmid DNA with rat HER2 ECD sequence and 4, rat HER2 ECD-IFNγ sequence; 5, Agarose gel electrophoresis images of transcribed mRNA and 6, total mRNA of rat HER2 ECD; 7, Agarose gel electrophoresis images of transcribed mRNA and 8, total mRNA of rat HER2 ECD-IFNγ.

Upon intramuscular administration, LNPs facilitate the delivery of encapsulated mRNA into human cells, and some LNPs directly transfect professional antigen-presenting dendritic cells (DCs). In transfected cells, mRNA is released and translated by ribosomes into antigenic proteins: cytosolic antigens are degraded by the immunoproteasome into short peptides, which are loaded onto MHC class I molecules for presentation to peptide-specific CD8^+^ T cells. In directly transfected DCs, antigenic proteins are additionally shuttled to the endolysosomal compartment via autophagy, where lysosomal degradation generates peptides that associate with MHC class II molecules to activate CD4^+^ T cells. Antigenic proteins secreted extracellularly can also be re-endocytosed into DCs for MHC class II presentation. CD4^+^ T cells then drive B cell activation and the production of HER2-specific antibodies. This sequential process of antigen processing, presentation and adaptive immune activation underpins the vaccine’s antitumor efficacy (**Figure 1A**) (38, 39). We engineered LNP formulations using a microfluidic mixing strategy to precisely encapsulate mRNA. The ethanol phase contained an optimized molar ratio of Dlin-MC3-DMA, DSPC, CHOL and DMG-PEG2000, which was meticulously combined with an aqueous phase containing purified mRNA, based on established methodologies with critical refinements (26, 27) (**Figure 1B****;** **Table 1**). High-purity mRNA was synthesized through *in vitro* transcription utilizing T7 RNA polymerase and meticulously engineered linearized plasmid templates (pcDNA3.1(+)-rat HER2 ECD and pcDNA3.1(+)-rat HER2 ECD-IFNγ). This process incorporated a 5’ Cap 1 structure and a precisely defined 3’ poly(A) tail of optimal length, followed by stringent purification (**Figure 1C**). The resulting mature mRNA constructs exhibited a canonical architecture, including the 5’ Cap 1, an optimized 5’ UTR, an open reading frame (ORF) with a signal peptide, a Kozak sequence, the HER2 ECD sequence, a 3’ UTR, and a poly(A) tail (**Figure 1B**). To confirm cross-species conservation of HER2 ECD, we performed NCBI BLASTP alignment of rat and human HER2 sequences. As shown in **Supplementary Table 2**, rat HER2 ECD shared 86.09% identity with human HER2 (99% query coverage, E-value = 0.0), validating epitope conservation for tolerance-breaking design. Comprehensive quality assessment confirmed the integrity and high purity of the mRNA through agarose gel electrophoresis, which displayed distinct, singular bands indicative of minimal degradation for both the precursor plasmid, linearized DNA, and the final mRNA products (**Figures 1D, E**). NanoCoulter analysis indicated that both rHER2 ECD mRNA-LNP and rHER2 ECD-IFNγ mRNA-LNP exhibited similar sizes and zeta potentials within the established range for efficacious LNP formulations. Moreover, these formulations showed low Span values and a low polydispersity index (PDI), reflecting a narrow particle size distribution. The quantification of encapsulation efficiency using the RiboGreen fluorescence assay consistently demonstrated values exceeding 80% for each mRNA-LNP formulation (**Table 2**). These analytical results collectively confirm the successful production of highly stable mRNA-LNP vaccines, which exhibit optimal nanoscale properties crucial for efficient delivery and immunogenicity.

**Table 2:** Physical properties of mRNA-LNPs.

Formulation	Average diameter (nm)	Span	Polydispersity index	Zeta potential (mV)	Encapsulation efficiency (%)
LNP	60.0 ± 1.0	0.22	0.17 ± 0.03	10.2 ± 1.5	–
rHER2 ECD mRNA-LNP	82.0 ± 4.1	0.60	0.16 ± 0.01	4.7 ± 0.8	80.3 ± 1.5
rHER2 ECD-IFNγ mRNA-LNP	90.2 ± 3.5	0.58	0.16 ± 0.01	4.5 ± 0.6	81.8 ± 2.6

### 3.2 HER2 ECD mRNA-LNP vaccines elicit striking tumor inhibition *in vivo*

**Figure 2:**
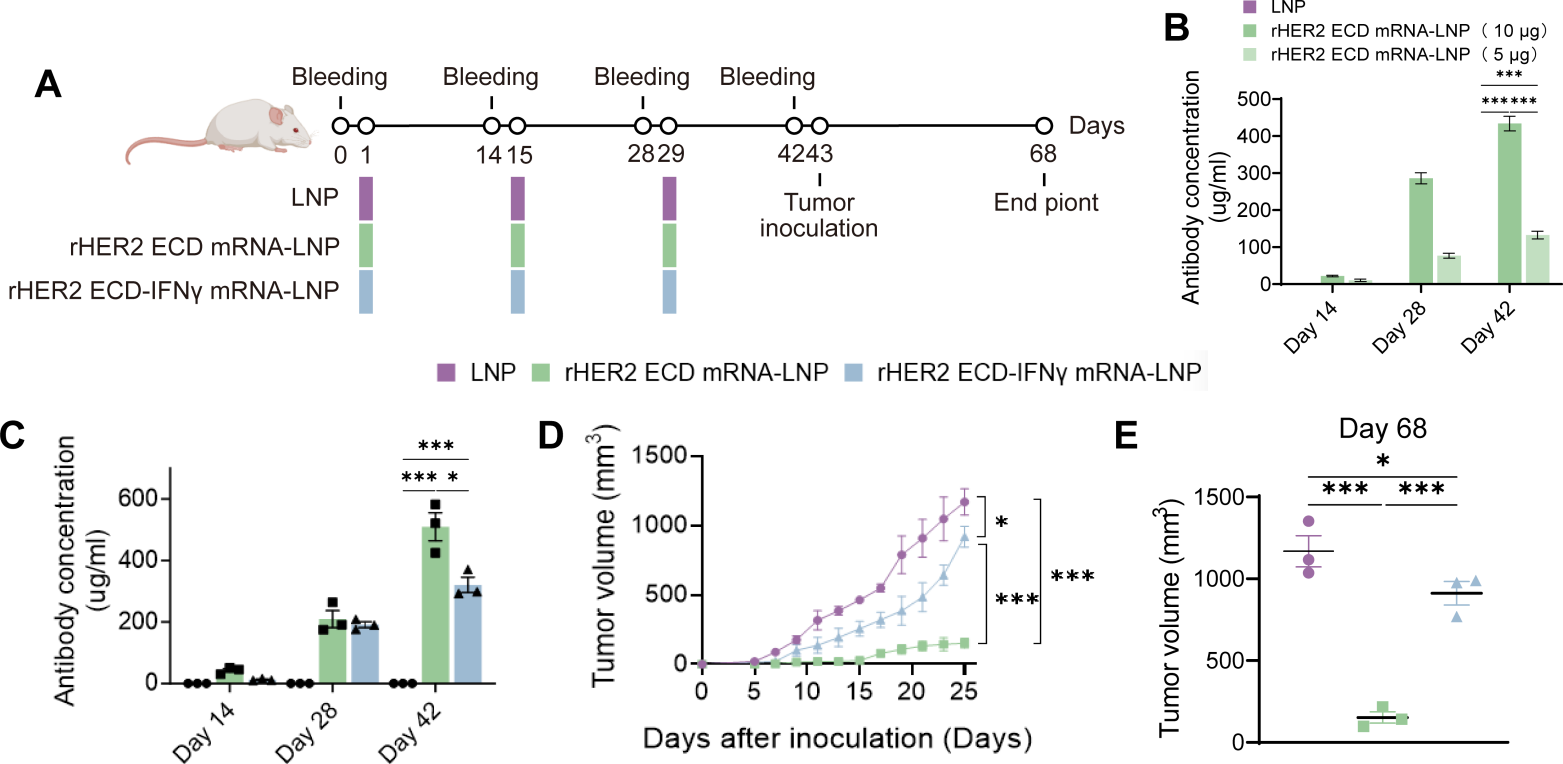
Therapeutic efficacy of HER2 ECD mRNA-LNP vaccines in 4T1-HER2 tumor-bearing mice. **(A)** Treatment schedules and dosing intervals of the 4T1-HER2 tumor model. **(B)** Serum anti-HER2 antibody kinetics after 5 μg or 10 μg rHER2 ECD mRNA-LNP vaccination. **(C)** Changes in serum HER2 antibody concentration over time after administration after rHER2 ECD mRNA-LNP or rHER2 ECD-IFNγ mRNA-LNP vaccination. **(D)** Growth curve of 4T1-HER2 tumors. **(E)** Representative images of tumor volume. N = 3. Data are presented as mean ± standard error of the mean (SEM); two-sided t-test. (*P < 0.05 and ***P < 0.001).

To assess the vaccine efficacy *in vivo*, four-to-six-week-old female BALB/c mice were randomly allocated into groups (N = 3 per group) and monitored using an established tumor-bearing mouse model, following the vaccination schedule depicted in **Figure 2A**. Animals received prime-boost vaccination with 10 μg mRNA-LNP through dose-finding studies establishing this regimen as optimal prime on day 1 followed by boosts on days 15 and 29 (**Figure 2B**). Comparative analysis of rHER2 ECD mRNA-LNP and rHER2 ECD-IFNγ mRNA-LNP demonstrated distinct antitumor efficacy, safety profiles, and immune responses. Notably, vaccination with rHER2 ECD mRNA-LNP resulted in significantly higher anti-HER2 antibody titers, indicative of robust B-cell-mediated immune activation (**Figure 2C**). To evaluate tumor suppression, immunized mice were subcutaneously inoculated with 4T1-HER2 breast cancer cells at Day 43. In alignment with robust immune protection, both vaccinated cohorts demonstrated significant tumor growth suppression compared to the rapid progression observed in unvaccinated controls. Notably, tumor burden quantification revealed a substantial inhibition rate of 87.0% ± 3.0% (*P* < 0.001) in the rHER2 ECD mRNA-LNP group, which significantly surpassed the modest 21.9% ± 6.1% (*P* < 0.05) inhibition observed in the rHER2 ECD-IFNγ mRNA-LNP group (**Figures 2D, E**).

### 3.3 HER2 ECD mRNA-LNP vaccines exhibit favorable systemic safety profiles

**Figure 3:**
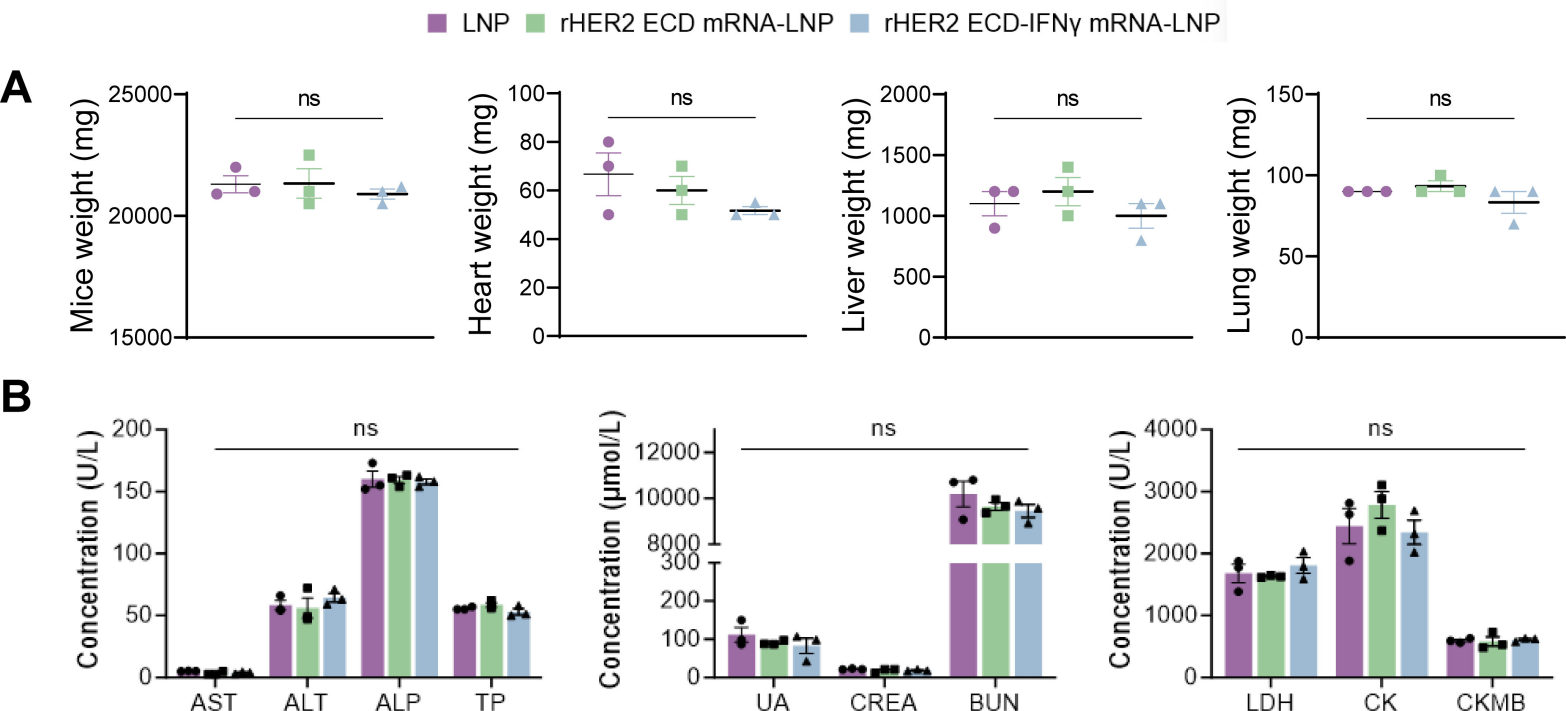
Safety profiles of HER2 ECD mRNA-LNP vaccines in 4T1-HER2 tumor-bearing mice. **(A)** Body, heart, liver and lung weights of mice. **(B)** Hepatic function, renal function and cardiac function on day 68. N = 3. Data are presented as mean ± SEM; two-sided t-test. No significant intergroup differences were observed.

Evaluation of systemic adverse reactions indicated that both vaccine formulations and the LNP vehicle were well-tolerated at the administered dose, as evidenced by stable body weights throughout the study and the absence of significant differences in heart, liver, or lung organ weights across all groups (**Figure 3A**). Comprehensive serum biochemical profiling at the study endpoint, which included markers of hepatic (AST, ALT, ALP), renal (BUN, CREA, UA), and cardiac (LDH, CK CKMB) function, as well as total protein levels, revealed no significant alterations indicative of functional impairment in any treatment cohort (**Figure 3B**). These comprehensive safety evaluations highlight the absence of treatment-related adverse effects within this model. The findings indicate that rHER2 ECD mRNA-LNP provides exceptional prophylactic antitumor efficacy, predominantly mediated by humoral immunity, while maintaining a highly favorable safety profile. Notably, the non-adjuvanted rHER2 ECD mRNA-LNP demonstrated significantly superior antitumor activity compared to its IFNγ-fused counterpart, warranting further mechanistic investigation.

### 3.4 rHER2 ECD mRNA-LNP promotes expansion of polyfunctional effector CD8^+^ T cells with enhanced cytokine secretion

**Figure 4:**
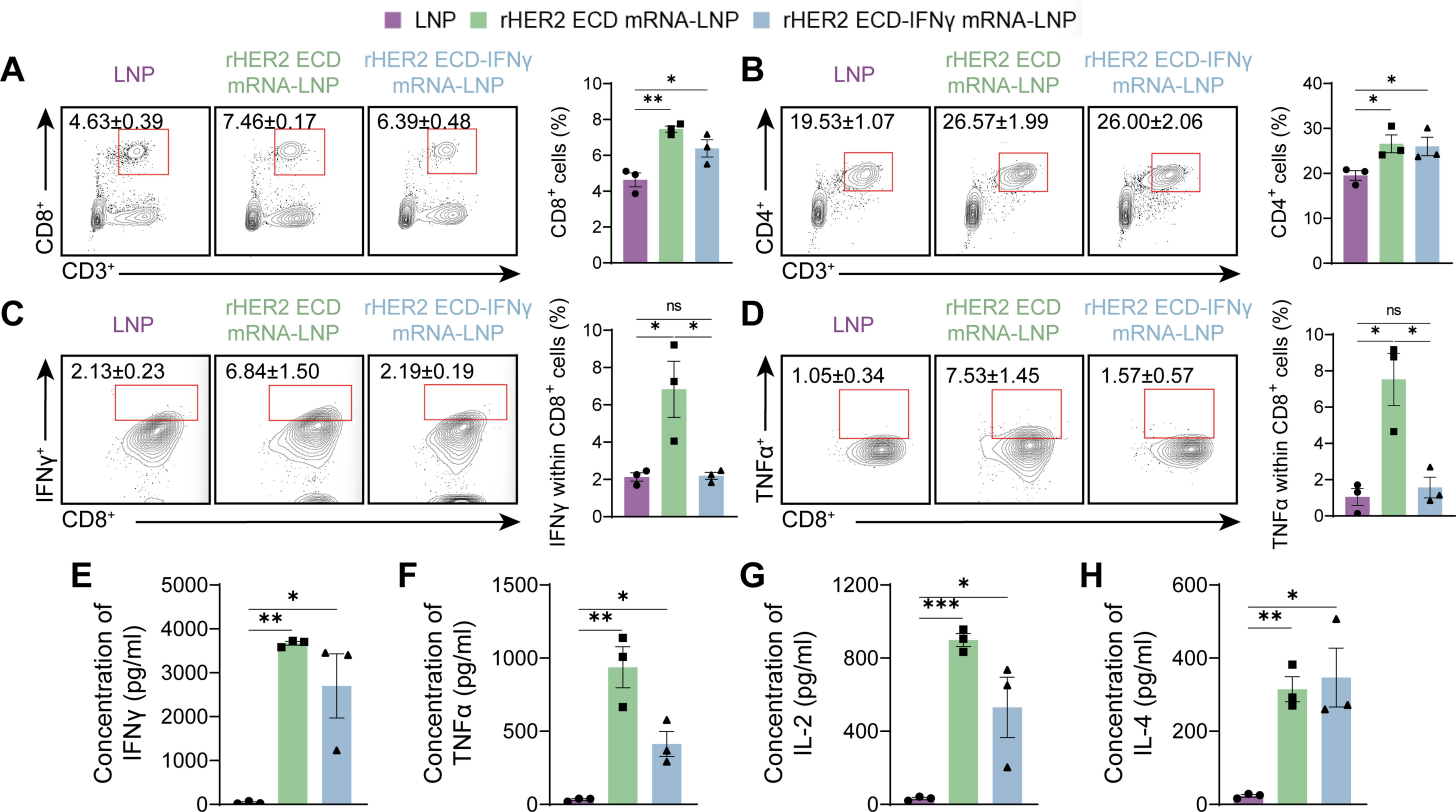
Vaccine-induced T cell responses and cytokine production. **(A)** Representative flow cytometry diagrams and percentages of CD8^+^ and **(B)** CD4^+^ T cells. **(C)** Percentages of IFNγ-positive cells within CD3^+^ CD8^+^ T cells. **(D)** Percentages of TNFα-positive cells within CD3^+^ CD8^+^ T cells. **(E)** IFNγ, **(F)** TNFα, **(G)** IL-2 and **(H)** IL-4 in mouse spleen culture supernatants. N = 3. Data are presented as mean ± SEM; two-sided t-test. (*P < 0.05, **P < 0.01, and ***P < 0.001).

To elucidate the cellular immune mechanisms underlying vaccine efficacy, we conducted flow cytometric analysis of splenocytes. Both vaccine groups showed a significant expansion of CD4^+^ and CD8^+^ T cells relative to LNP controls, with the rHER2 ECD mRNA-LNP group exhibiting substantially higher T cell frequencies (**Figures 4A, B**). Further analysis revealed that rHER2 ECD mRNA-LNP vaccination significantly increased the frequency of CD8^+^ T cells co-producing the effector cytokines IFNγ and TNFα, achieving levels of 6.8% ± 1.5% (*P* < 0.05) and 7.5% ± 1.5% (*P* < 0.05), respectively, which were significantly higher than control levels. In a noteworthy observation, the administration of rHER2 ECD-IFNγ mRNA-LNP did not result in a significant enhancement of effector cytokine production (**Figures 4C, D**). Unstimulated splenocytes from all groups confirmed negligible background cytokine expression across treatment groups, validating that the signals reflect bona fide T cell functional potential upon stimulation (**Supplementary Figures 1A, B**). Corroborating these results, ELISA analysis of secreted cytokines revealed markedly elevated levels of IFNγ (**Figure 4E**) and TNFα (**Figure 4F**) in the supernatant of HER2 antigen-stimulated splenocytes from the rHER2 ECD mRNA-LNP group. Additionally, this group demonstrated a substantial increase in IL-2 (**Figure 4G**) and IL-4 (**Figure 4H**) secretion, suggesting robust CD8^+^ T cell effector function alongside a concurrent Th2-skewed immune response. Additional experiments involving unstimulated splenocytes and 6-hour stimulations consistently showed low cytokine levels across all groups, further validating antigen-specific activation (**Supplementary Figure 2**). In contrast, such alterations were less prevalent in the rHER2 ECD-IFNγ mRNA-LNP group. These findings indicate that although the IFNγ fusion facilitated quantitative T cell expansion, it significantly compromised qualitative effector differentiation, resulting in an expansion of T cells that were functionally impaired.

### 3.5 rHER2 ECD mRNA-LNP induces durable central/effector memory T cell compartments

**Figure 5:**
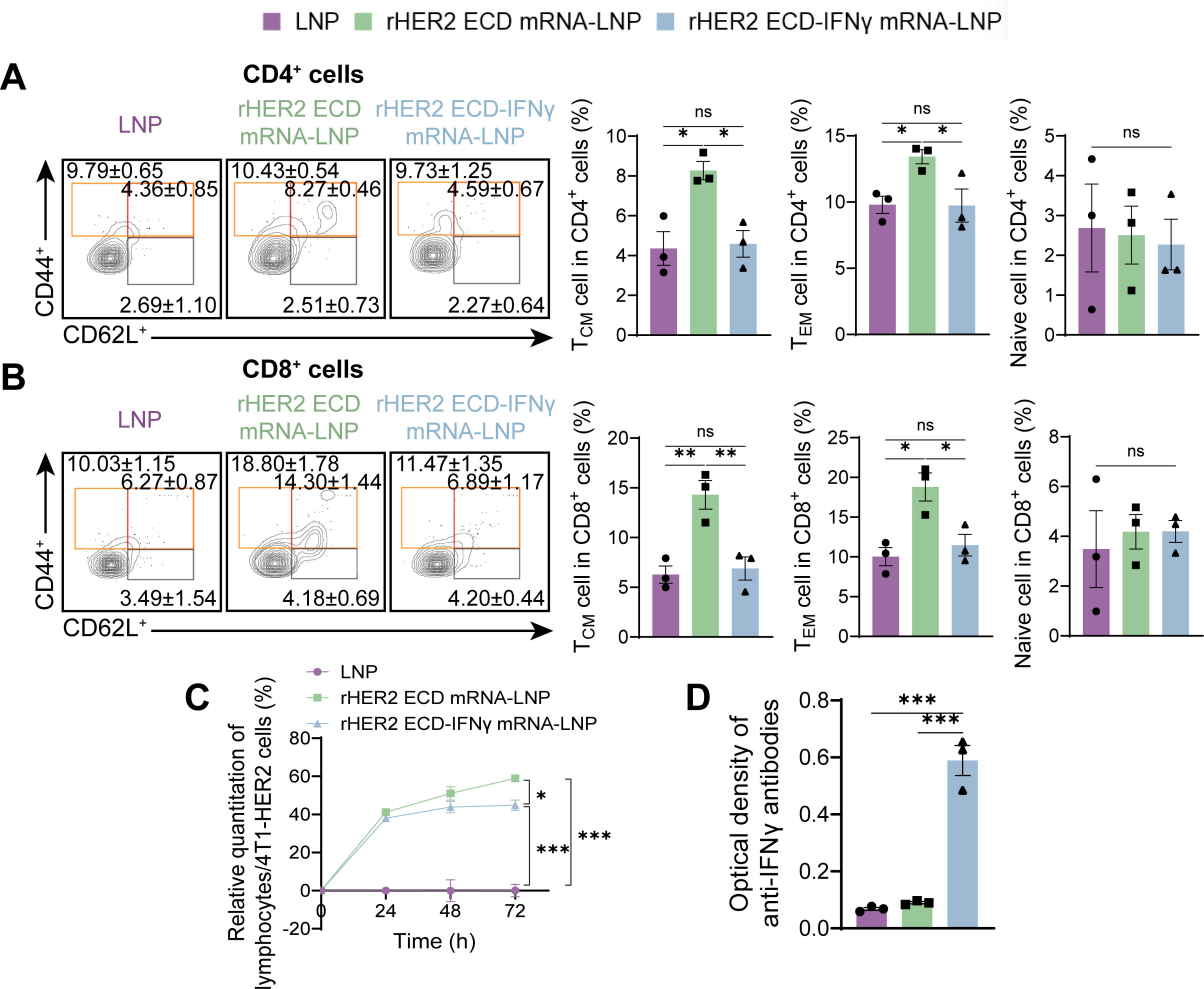
Memory T cell subsets and antitumor activity. **(A)** Representative flow cytometry plots of T_CM_ cells (CD44^+^ CD62L^+^), T_EM_ cells (CD44^+^) and naive cells (CD44^-^ CD62L^+^) within CD4^+^ and **(B)** CD8^+^ T cells. **(C)** Rate of inhibition of 4T1-HER2 cells by vaccinated mouse splenic lymphocytes. **(D)** Concentration of IFNγ antibody in serum after administration. N = 3. Data are presented as mean ± SEM; two-sided t-test. (*P < 0.05, **P < 0.01, and ***P < 0.001).

Evaluation of immunological memory, a critical component of enduring antitumor immunity, showed that rHER2 ECD mRNA-LNP vaccination substantially increased both central memory (T_CM_) and effector memory (T_EM_) T cell subsets within the CD4^+^ and CD8^+^ compartments, while preserving the naïve T cell population. In contrast, the rHER2 ECD-IFNγ mRNA-LNP elicited minimal differentiation of memory T cells (**Figures 5A, B**). Building upon these phenotypic findings, we proceeded to evaluate functional cytotoxicity through an *in vitro* coculture assay involving splenocytes and 4T1-HER2 tumor cells. Splenocytes derived from mice immunized with either the rHER2 ECD mRNA-LNP or the rHER2 ECD-IFNγ mRNA-LNP demonstrated a time-dependent increase in cytotoxic activity, culminating in maximal lysis rates of 58.9% ± 1.7% (*P* < 0.01) and 44.9% ± 2.7% (*P* < 0.01), respectively, at 72 hours (**Figure 5C**). Collectively, these findings indicate that the rHER2 ECD mRNA-LNP vaccination surpassed its IFNγ-fused counterpart in eliciting functional effector responses, including cytokine production and cytotoxicity, as well as memory T cell differentiation and a Th2 bias. To elucidate the mechanistic underpinnings of this disparity, we quantified anti-IFNγ antibodies. Notably, immunization with the rHER2 ECD-IFNγ mRNA-LNP resulted in significant seroconversion of anti-IFNγ antibodies, a phenomenon not observed in the LNP control or rHER2 ECD mRNA-LNP groups (**Figure 5D**). This suggests that antibody-mediated neutralization of the fused cytokine effectively nullified its intended immunostimulatory function. Therefore, while the fusion of IFNγ initially promoted T cell expansion, it simultaneously triggered the production of neutralizing antibodies that hindered effector maturation and memory formation, ultimately diminishing the vaccine’s efficacy.

### 3.6 Combination of rHER2 ECD mRNA-LNP and immune checkpoint inhibitor improved antitumor efficacy

**Figure 6:**
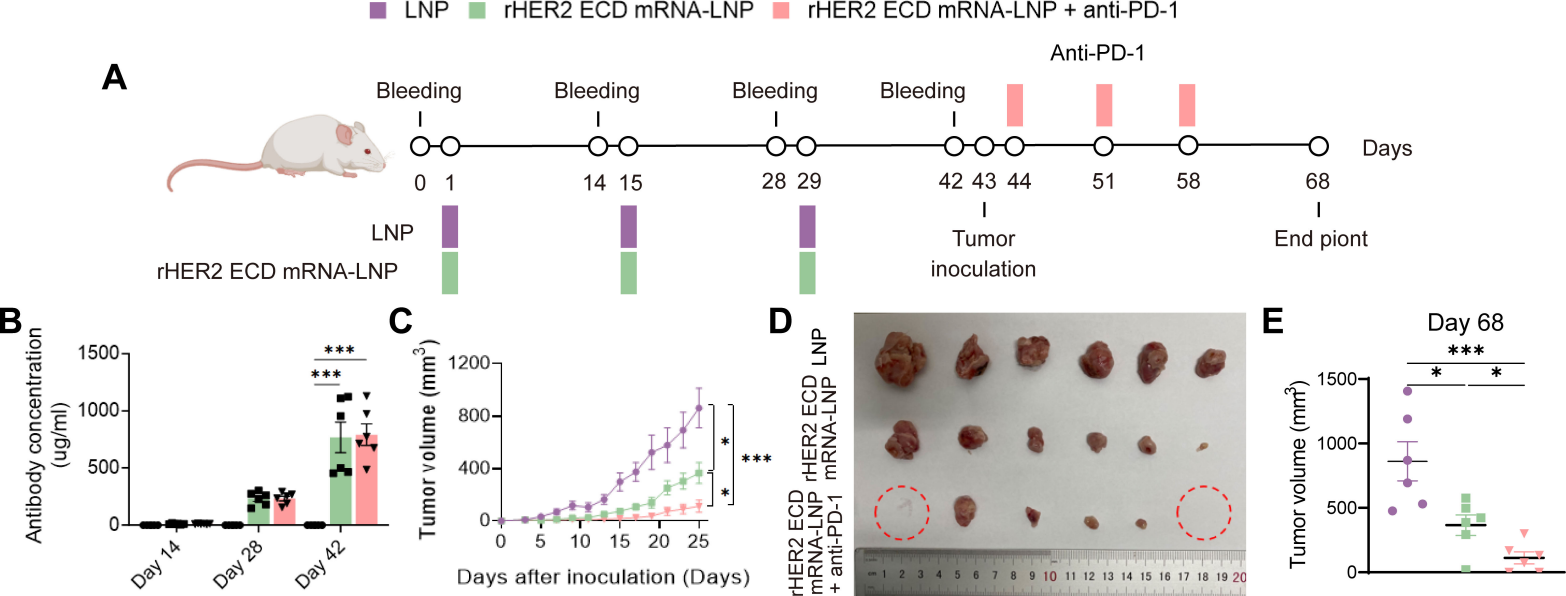
Antitumor efficacy of combination therapy. **(A)** Treatment schedules and dosing intervals of the 4T1-HER2 tumor model. **(B)** Changes in serum HER2 antibody concentration over time after administration after rHER2 ECD mRNA-LNP vaccination, with and without anti-PD-1. **(C)** Growth curve of 4T1-HER2 tumors. **(D)** Representative images of tumor tissue. **(E)** Tumor volume. N = 6;Data are presented as mean ± SEM. Two-sided t-test. (*P < 0.05 and ***P < 0.001).

Building on the demonstrated effectiveness of rHER2 ECD mRNA-LNP in inducing potent T cell effector functions and durable memory, we proceeded to explore its potential in combination with immune checkpoint blockade (ICB). Given the limited clinical success of PD-1 inhibitors in breast cancer, which is often due to primary resistance mechanisms such as inadequate T cell priming (40, 41) and a highly immunosuppressive TME (42), we hypothesized that our vaccine could counteract these challenges by facilitating robust antigen-specific T cell activation. To evaluate this synergy hypothesis, mice immunized with rHER2 ECD mRNA-LNP vaccination were inoculated with 4T1-HER2 tumor cells (N = 6). One day after inoculation, mice were administered with three intraperitoneal doses of anti-PD-1 mAb at 7-day intervals (**Figure 6A**). Notably, the anti-PD-1 mAb utilized in this study possesses L234A/L235A (LALA) mutations within its human IgG1 Fc region. These mutations significantly abrogate antibody-dependent cellular cytotoxicity (ADCC) and complement-dependent cytotoxicity (CDC) by impairing interactions with Fcγ receptors and C1q (43, 44). Consequently, this rules out the possibility that alterations in T cell numbers or functions arise from antibody-mediated depletion of PD-1^+^ T cells. The vaccination resulted in a dose-dependent increase in anti-HER2 antibody titers following the third immunization (**Figure 6B**). The combination therapy significantly inhibited tumor growth, achieving an inhibition rate of 87.0% ± 5.4% (*P* < 0.001) compared to LNP controls, and notably exceeding the 57.5% ± 9.2% (*P* < 0.05) inhibition observed with rHER2 ECD mRNA-LNP monotherapy (**Figure 6C**). Importantly, with the combination regimen, one-third of tumors (2/6) failed to establish. This outcome was not observed in any subject in the vaccine monotherapy cohort (**Figures 6D, E**).

### 3.7 Mechanisms underlying synergy: T cell redistribution, tissue-resident memory T enrichment, and qualitative TME remodeling in early-stage tumors

**Figure 7:**
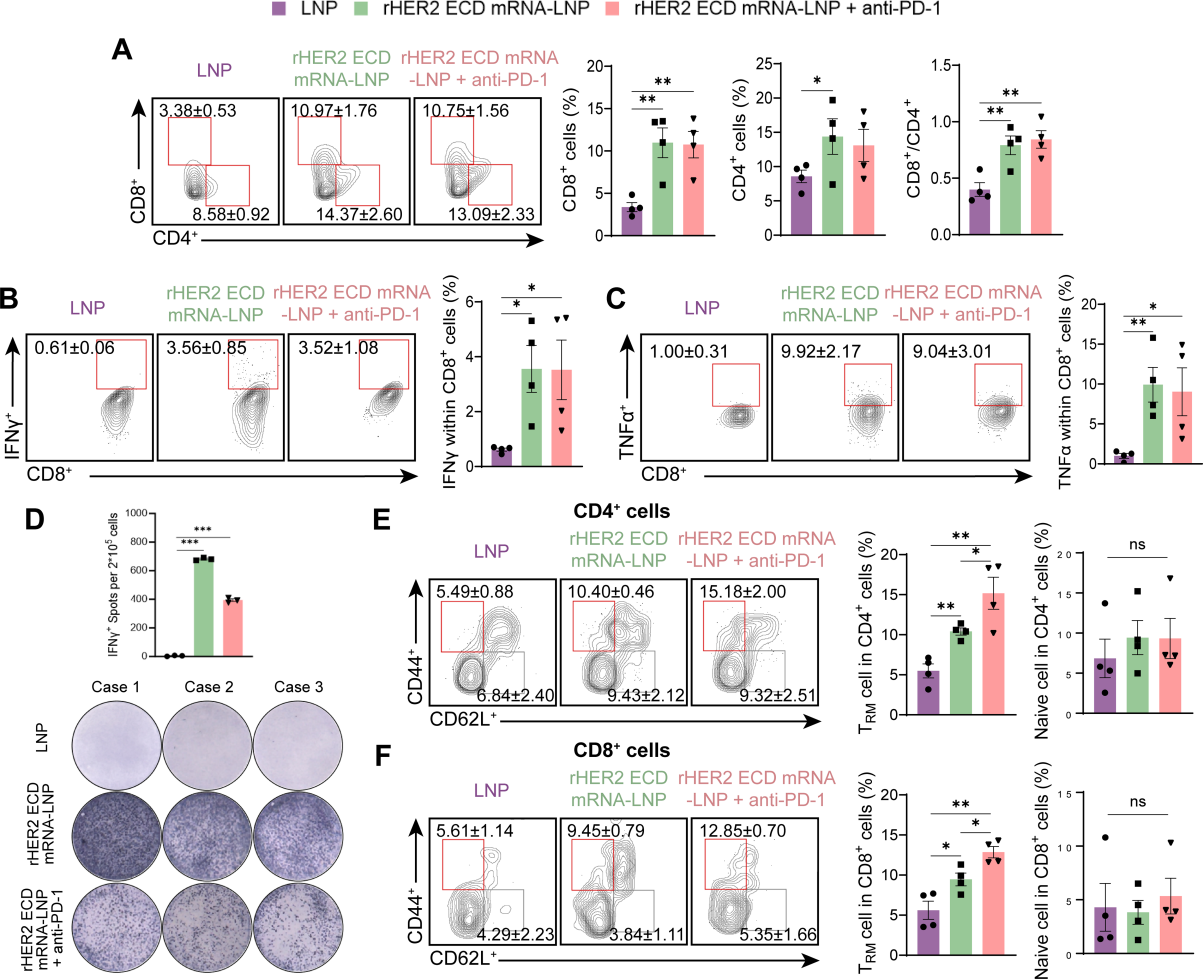
Tumor-infiltrating lymphocytes and T_RM_ cells. **(A)** Representative flow cytometry diagrams and percentages of CD4^+^ and CD8^+^ T cells on day 68 in tumor tissues (N = 4). **(B)** Representative flow cytometry diagrams and percentages of CD8^+^ T cells expressing IFNγ and **(C)** TNFα on day 68 in tumor tissues (N = 4). **(D)** ELISpot images and spot nos. of IFNγ-secreting T cells within splenocyte of vaccinated mice (N = 3). **(E)** Representative flow cytometry plots of T_RM_ cells (CD44^+^ CD62L^-^) and naive cells (CD44^-^ CD62L^+^) within CD4^+^ and **(F)** CD8^+^ T cells in tumor tissues (N = 4). Data are presented as mean ± SEM. Two-sided t-test; Data for CD4^+^ T cells in **(A)** were analyzed by one-tailed Student’s t-test. (*P < 0.05, **P < 0.01, and ***P < 0.001).

Analysis of tumor-infiltrating lymphocytes (TILs) revealed that combining rHER2 ECD mRNA-LNP with anti-PD-1 mAb did not significantly increase overall tumor-infiltrating CD4^+^ or CD8^+^ T cell frequencies beyond monotherapy levels (**Figure 7A**). Although CD8^+^ T cells from combination-treated mice showed significantly elevated IFNγ and TNFα production relative to controls, these levels did not differ statistically from the monotherapy group (**Figures 7B, C**). No significant differences in cytokine-positive T cell frequencies were observed among the unstimulated splenocytes from different groups (**Supplementary Figures 1C, D**). We subsequently assessed the frequency of IFNγ-secreting T cells in splenocytes using ELISpot assay following ex vivo HER2 antigen restimulation. While the vaccine monotherapy group exhibited an increased number of IFNγ-secreting T cells, the combination treatment group showed reduced activity relative to the monotherapy group (**Figure 7D**).

Notably, this reduction was not due to impaired immune activation, but rather reflects the spatial redistribution of HER2-specific T cells from the spleen to the TME. Given that the LALA-mutated anti-PD-1 mAb precludes antibody-mediated T cell depletion (43, 44), the decrease in splenic IFNγ^+^ cells can be attributed to the enhanced migration of vaccine-primed T cells to tumors, as evidenced by the notable enrichment of tissue-resident memory T (T_RM_) cells in the TME of the combination group (**Figures 7E, F**). As T_RM_ cells primarily originate from peripheral effector T cells (e.g., splenocytes) (45), their accumulation in the TME inevitably depletes the splenic reservoir of HER2-specific T cells available for immediate IFNγ secretion, directly explaining the lower IFNγ activity detected in splenic ELISpot assays. In fact, the combination therapy selectively and significantly expanded the population of T_RM_ cells, a critical subset necessary for sustaining long-term local immunosurveillance and mounting rapid cytotoxic responses upon antigen re-encounter. In the combination group, T_RM_ frequencies in the TME reached 15.2% ± 2.0% (*P* < 0.01) among CD4^+^ T cells and 12.9% ± 0.7% (*P* < 0.01) among CD8^+^ T cells (**Figures 7E, F**). These results indicate that PD-1 blockade enhances antitumor efficacy not merely via quantitative increases in T cell infiltration, but through qualitative modulation of the early-stage TME, whereby significant T_RM_ cell enrichment promotes sustained T cell functionality and persistence within the tumor niche.

Collectively, the rHER2 ECD mRNA-LNP vaccine synergizes with anti-PD-1 therapy by addressing complementary limitations in antitumor immunity. The combination of vaccine-mediated antigen-specific T cell activation and checkpoint inhibition-driven reversal of T cell differentiation remodeling yields a potentiated immune response. This synergy is mechanistically supported by the establishment of a durable T_RM_ pool, which maintains active immunological surveillance within the TME and enables superior tumor control.

## 4 Discussion

Targeting HER2, an oncogenic driver overexpressed in multiple cancers, presents challenges owing to HER2-specific immune tolerance and TME-mediated immune suppression. This study demonstrates the successful engineering and robust immunogenicity of an mRNA-LNP vaccine encoding the rat HER2 ECD. Our data indicate that the rHER2 ECD mRNA-LNP elicits profound antitumor immunity, characterized by strong humoral responses, activation of functional effector CD8^+^ T cells, and the formation of durable memory T cells. The comprehensive inhibition of HER2-overexpressing tumor growth *in vivo* highlights the effectiveness of this vaccination strategy.

The remarkable prophylactic efficacy of the rHER2 ECD mRNA-LNP vaccine observed in our study highlights its potential to address critical unmet clinical needs in the management of HER2-positive malignancies. Although adjuvant mAb therapies plus chemotherapies have significantly improved patient survival outcomes, their clinical utility is constrained by the frequent emergence of resistance mechanisms (46, 47). In sharp contrast, our mRNA-LNP vaccine elicits a robust, durable polyclonal antibody response: unlike mAbs that target a single epitope, the polyclonal reactivity induced by the full-length ECD encoded in our vaccine enables multi-epitope targeting, thereby mitigating the risk of tumor immune escape via antigen downregulation or epitope masking (48). Notably, this study underscores the unique value of mRNA-LNP platforms for secondary prophylaxis, a critical strategy to prevent disease recurrence following curative-intent treatment for early-stage HER2-positive breast cancer. MRD is the primary driver of relapse in this setting, and our data indicate that the mRNA-LNP platform supports sustained antigen presentation and the generation of long-lived central and effector memory T cell populations. This durable immune reserve is theoretically poised to detect and eliminate micrometastatic foci that emerge long after the discontinuation of passive antibody therapy (49).

In our investigation of the rHER2 ECD-IFNγ mRNA-LNP vaccine as a single agent, the in-frame fusion design was predicated on seminal studies demonstrating that antigen-coupled IFNγ enhances Th1-type cellular immunity (50, 51). Contrary to our expectations, immunization with this construct induced the production of anti-IFNγ neutralizing antibodies in mice, which correlated with diminished vaccine efficacy. This phenomenon aligns with the literature indicating that immune perturbations, including sustained cytokine exposure, can break tolerance and elicit specific autoantibodies (52, 53). Critically, our data indicated that the fusion vaccine mediated IFNγ secretion, but its bioactivity was subsequently abrogated by antibody-mediated neutralization. This was evidenced by the correlation between anti-IFNγ antibody titers and defects in effector CD8^+^ T cell differentiation and memory (T_CM_/T_EM_) generation. Therefore, the key barrier is not defective secretion, but the induction of neutralizing antibodies. This underscores the necessity for future vaccine optimization to preserve cytokine bioactivity while enhancing memory T cell formation.

Moving beyond prophylaxis to explore therapeutic applications, we evaluated a combination strategy comprising the rHER2 ECD mRNA-LNP vaccine and anti-PD-1 mAb. Early administration of PD-1 blockade was employed to mitigate T cell exhaustion, potentially driven by the rapid upregulation of PD-L1 in the evolving TME, thus maintaining the functionality of vaccine-primed T cells (54, 55). The combined therapy achieved a significantly superior antitumor effect compared to single-agent therapy. Mechanistically, this synergy is associated with a marked increase in T_RM_ cells within the tumor. T_RM_ cells can reside in tumor tissues for a long time, providing continuous immune surveillance and rapid cytotoxic responses upon antigen re-encounter (56). Unlike the deeply entrenched immunosuppressive microenvironment of advanced established tumors that hinders conventional T cell responses, the prophylactic and early intervention settings employed in this study likely allow PD-1 blockade to prevent the initial establishment of local immunosuppression. In our combination regimen, the vaccine induces HER2-specific T cells, while PD-1 blockade counters adaptive immune resistance, jointly fostering a TME conducive to T_RM_ cell infiltration and accumulation. Consequently, this synergistic remodeling effectively converts the typically “cold” or immune-excluded microenvironment into an inflamed, “hot” phenotype, providing a mechanistic rationale for deploying this vaccine as a therapeutic agent in combination with checkpoint inhibitors for active disease (57).

Beyond targeting HER2, this research introduces a broadly applicable mRNA-LNP platform for targeting tumor antigens. Key principles such as LNP optimization, antigen design, avoidance of counterproductive immunogenicity, and T_RM_-mediated synergy with ICB provide a framework applicable to a variety of solid tumors. The rapid encoding capability of mRNA technology facilitates versatile and personalized cancer immunotherapy. Future research should investigate the integration of existing HER2-targeted biologics with radiochemotherapy, identify predictive biomarkers associated with humoral and cellular immunity, and develop strategies to counteract antigen escape via neoantigen incorporation.

There are some limitations in this study. First, immune responses against rat HER2 in mice may not fully recapitulate those targeting human HER2 in patients, despite high sequence homology. Second, we did not visualize cytokine production during direct T cell-tumor contact. Third, as our study utilized a prophylactic model, the tumors likely lacked dense fibrotic stroma and deeply entrenched immunosuppressive networks typical of advanced clinical disease. Thus, further validation in established therapeutic models is required to confirm the efficacy of the strategy against such barriers. Fourth, while we observed T_RM_ enrichment, the precise molecular mechanisms underlying vaccine-induced memory differentiation warrant deeper investigation using single-cell transcriptomics or TCR sequencing. Finally, the role of ADCC mediated by vaccine-induced antibodies and Natural Killer (NK) cells requires further investigation.

## 5 Conclusion

In summary, we have developed an optimized rHER2 ECD mRNA-LNP vaccine that effectively circumvents immune tolerance, inducing robust polyfunctional CD8^+^ T cells and long-lasting T_CM_ and T_EM_ T cells, resulting in superior prophylactic tumor control. Notably, the combination of this vaccine with anti-PD-1 therapy collectively achieved superior tumor rejection, primarily through the qualitative enrichment of T_RM_ cells within the TME rather than through an increase in T cell numbers. These findings demonstrate a potent and safe mRNA-LNP platform that induces coordinated humoral and cellular immunity against HER2, offering mechanistic insights into T_RM_-mediated synergy for transformative immunotherapy in prophylactic and early-stage HER2-positive cancer settings.

## Data Availability

The original contributions presented in the study are included in the article/**Supplementary Material**. Further inquiries can be directed to the corresponding authors.
