# Supplementary material for: Robust CD8+ T cell responses induced by an mRNA-LNP vaccine encoding rat HER2 extracellular domain confer prophylactic tumor protection
Source: Front Immunol. 2026 Apr 1;17:1737558. doi: 10.3389/fimmu.2026.1737558 (PMC13079660; doi:10.3389/fimmu.2026.1737558)
Supplement: Supplementary file 1 [file Image1.pdf]

Supplementary Figures

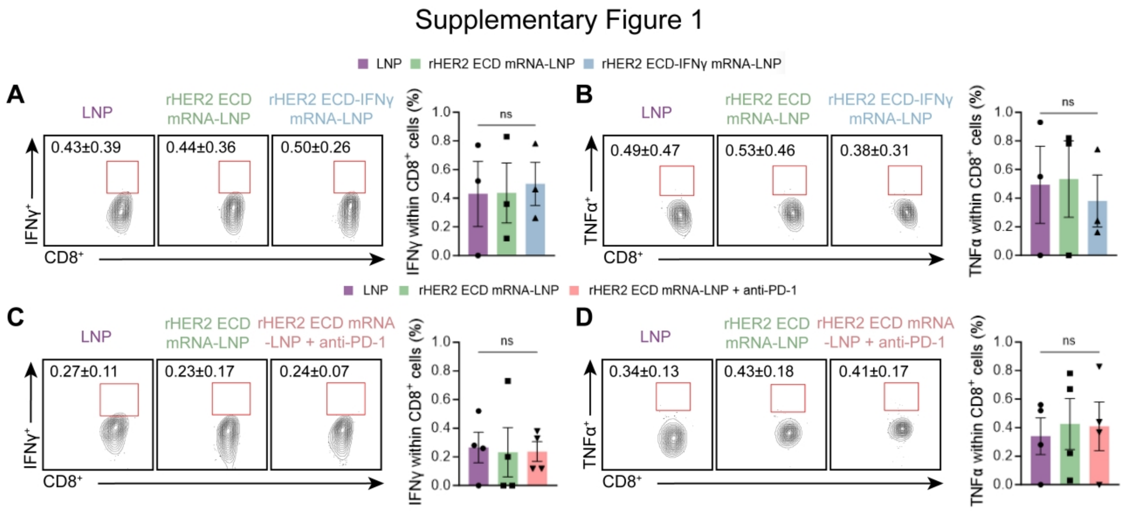

SUPPLEMENTARY FIGURE 1 Baseline cytokine expression in unstimulated splenocytes. **(A)** Representative flow cytometry dot plots of IFNγ^{+} CD8^{+} T cells, and **(B)** TNFα^{+} CD8^{+} T cells in unstimulated splenocytes from LNP control, rHER2 ECD mRNA-LNP, and rHER2 ECD-IFNγ mRNA-LNP groups (N = 3). **(C)** Representative flow cytometry dot plots of IFNγ^{+} CD8^{+} T cells, and **(D)** TNFα^{+} CD8^{+} T cells in unstimulated splenocytes from LNP control, rHER2 ECD mRNA-LNP, and rHER2 ECD mRNA-LNP + anti-PD-1 groups (N = 4). Data are presented as mean ± SEM; two-sided t-test. No significant intergroup differences were observed.

Supplementary Figure 2

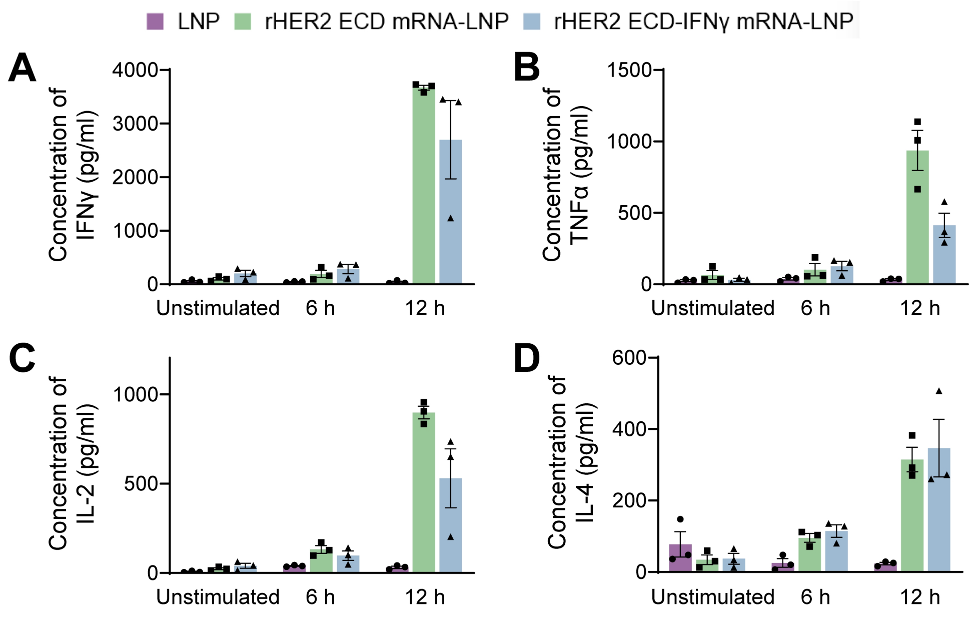

SUPPLEMENTARY FIGURE 2 Expression levels of cytokines in splenocyte culture supernatants under different stimulation conditions.

Following culture without stimulation or with recombinant rat HER2 protein for 6 h or 12 h, splenocytes from vaccinated mice were assessed for supernatant concentrations of **(A)** IFNγ, **(B)** TNFα, **(C)** IL-2, and **(D)** IL-4 by ELISA. N = 3. Data are presented as mean ± SEM.
