# Supplementary material for: Robust CD8+ T cell responses induced by an mRNA-LNP vaccine encoding rat HER2 extracellular domain confer prophylactic tumor protection
Source: Front Immunol. 2026 Apr 1;17:1737558. doi: 10.3389/fimmu.2026.1737558 (PMC13079660; doi:10.3389/fimmu.2026.1737558)
Supplement: Supplementary file 2 [file Table1.docx]

# **Supplementary Tables**

**SUPPLEMENTARY TABLE 1 Summary of nucleotide sequences**

| **Title** | **Nucleotide Sequences** |
| --- | --- |
| **rat HER2 ECD in ORF**  **(1896 bp)** | ACCCAGGTGTGCACCGGCACAGATATGAAGCTGAGACTGCCTGCCTCCCCCGAGACACACCTGGATATGCTGAGACACCTGTACCAGGGCTGTCAGGTGGTGCAGGGCAACCTGGAGCTGACATACGTGCCCGCCAACGCCTCCCTGAGCTTCCTGCAGGACATCCAGGAGGTGCAGGGCTACATGCTGATCGCCCACAACCAGGTGAAGAGGGTGCCCCTGCAGAGACTGAGGATCGTGAGAGGCACCCAGCTGTTCGAGGACAAGTACGCCCTGGCCGTGCTGGACAACAGGGACCCTCAGGATAATGTGGCCGCCTCCACACCCGGCAGAACACCCGAGGGCCTGAGGGAGCTGCAGCTGAGGAGCCTGACAGAGATCCTGAAGGGCGGCGTGCTGATCAGAGGCAACCCCCAGCTGTGCTACCAGGACATGGTGCTGTGGAAGGACGTGTTTAGAAAGAATAACCAGCTGGCCCCTGTGGATATCGATACCAACAGGAGCAGGGCCTGCCCCCCCTGTGCCCCTGCTTGTAAGGACAACCACTGTTGGGGCGAGTCCCCCGAGGACTGTCAGATCCTGACCGGCACCATCTGTACATCCGGCTGCGCCAGATGCAAGGGCAGGCTGCCTACCGACTGTTGTCACGAGCAGTGTGCCGCCGGCTGTACCGGCCCTAAGCACAGCGATTGTCTGGCCTGCCTGCACTTTAACCACAGCGGCATCTGCGAGCTGCACTGCCCTGCCCTGGTGACCTACAATACAGACACATTTGAGAGCATGCACAATCCTGAGGGCAGATACACCTTCGGCGCCAGCTGTGTGACAACCTGTCCTTACAATTACCTGAGCACCGAGGTGGGCAGCTGCACACTGGTGTGTCCCCCCAACAACCAGGAGGTGACCGCCGAGGACGGCACCCAGAGGTGCGAGAAGTGTTCCAAGCCCTGCGCCAGGGTGTGCTACGGCCTGGGAATGGAGCACCTGAGAGGCGCCAGGGCCATCACCAGCGACAATGTGCAGGAGTTCGACGGCTGCAAGAAGATCTTTGGCTCCCTGGCCTTTCTGCCTGAGAGCTTCGATGGCGACCCTTCCAGCGGCATCGCCCCCCTGAGGCCAGAGCAGCTGCAGGTGTTCGAGACCCTGGAGGAGATCACAGGCTACCTGTACATCTCCGCCTGGCCCGACTCCCTGAGGGATCTGTCCGTGTTCCAGAATCTGAGAATCATCAGAGGCAGGATCTTGCACGACGGCGCCTACAGCCTGACACTGCAGGGCCTGGGCATCCACAGCCTGGGCCTGAGAAGCCTGAGAGAGCTGGGCAGCGGCCTGGCCCTGATCCACAGAAACGCCCACCTGTGCTTTGTGCACACAGTGCCCTGGGACCAGCTGTTCAGGAATCCCCACCAGGCCCTGCTGCACAGCGGCAACAGGCCTGAGGAGGATCTGTGTGTGTCCAGCGGCCTGGTGTGCAACAGCCTGTGCGCCCACGGCCACTGCTGGGGACCTGGACCTACCCAGTGCGTGAACTGCAGCCACTTCCTGAGGGGCCAGGAGTGCGTGGAGGAGTGTAGGGTGTGGAAGGGCCTGCCTAGGGAGTACGTGAGCGACAAGAGGTGTCTGCCTTGTCACCCTGAGTGCCAGCCTCAGAACTCCAGCGAGACATGTTTCGGCTCCGAGGCCGACCAGTGCGCCGCTTGTGCCCACTACAAGGATTCCAGCAGCTGCGTGGCCAGGTGTCCCTCCGGCGTGAAGCCCGACCTGTCCTACATGCCTATCTGGAAGTACCCCGATGAGGAGGGCATCTGCCAGCCCTGCCCCATCAACTGTACACACTCCTGCGTGGATCTGGACGAGAGAGGCTGTCCTGCCGAGCAGAGAGCCTCCCCTGTGACA |
| **IFNγ in ORF**  **(399 bp)** | CACGGCACAGTGATCGAGTCCCTGGAGAGCCTGAACAACTACTTCAACTCCTCCGGCATCGACGTGGAGGAGAAGTCCCTGTTCCTGGATATCTGGAGAAATTGGCAGAAGGATGGCGACATGAAGATCCTGCAGAGCCAGATCATCAGCTTCTACCTGAGGCTGTTTGAGGTGCTGAAGGATAACCAGGCCATCTCCAATAATATCAGCGTGATCGAGAGCCACCTGATCACCACCTTCTTTTCCAATAGCAAGGCCAAGAAGGATGCCTTCATGTCCATCGCCAAGTTCGAGGTGAACAATCCCCAGGTGCAGAGACAGGCCTTCAATGAGCTGATCAGAGTGGTGCACCAGCTGCTGCCCGAGAGCTCCCTGAGGAAGAGGAAGAGGTCCAGGTGC |
|  | **Linkers in ORF** |
| **T7 promoter (19 bp)** | TAATACGACTCACTATAGG |
| **Kozak (6 bp)** | GCCACC |
| **Signal peptide**  **(66 bp)** | ATGGAGCTGGCGGCCTTGTGCCGCTGGGGGCTCCTCCTCGCCCTCTTGCCCCCCGGAGCCGCGAGC |
| **Poly-G linker**  **(15 bp)** | GGCGGCGGCGGCAGC |
| **5'UTR (46 bp)** | CTGAAACACGGTGGAGAGTTTATTGCAAAATAACGCGTCCATTCGA |
| **3'UTR (116 bp)** | TGATAGGCTGGAGCCTCGGTGGCCATGCTTCTTGCCCCTTGGGCCTCCCCCCAGCCCCTCCTCCCCTTCCTGCACCCGTACCCCCGTGGTCTTTGAATAAAGTCTGAGTGGGCGGC |

**SUPPLEMENTARY TABLE 2 Top 10 sequences with significant alignments**

| **Accession** | **Scientific Name** | **Query Cover** | **E value** | **Per. Ident** | **Acc. Len** |
| --- | --- | --- | --- | --- | --- |
| NP_001369723.1 | Homo Sapiens | 99% | 0.0 | 86.09% | 3730 |
| NP_001369725.1 | Homo Sapiens | 99% | 0.0 | 86.09% | 4470 |
| NP_004439.2 | Homo Sapiens | 99% | 0.0 | 86.09% | 4371 |
| NP_001369729.1 | Homo Sapiens | 99% | 0.0 | 86.09% | 4262 |
| NP_001369721.1 | Homo Sapiens | 99% | 0.0 | 86.09% | 3606 |
| NP_001369722.1 | Homo Sapiens | 99% | 0.0 | 86.09% | 4521 |
| NP_001369726.1 | Homo Sapiens | 99% | 0.0 | 86.09% | 4401 |
| NP_001369732.1 | Homo Sapiens | 99% | 0.0 | 85.93% | 4515 |
| NP_001369727.1 | Homo Sapiens | 99% | 0.0 | 86.09% | 4515 |
| NP_001276866.1 | Homo Sapiens | 99% | 0.0 | 85.93% | 4458 |
